# Supplementary material for: Are carbon emissions trading and green financial instruments synergistic? -Comprehensive quantitative research based on content analysis
Source: PLoS One. 2024 Mar 7;19(3):e0298601. doi: 10.1371/journal.pone.0298601 (PMC10919601; doi:10.1371/journal.pone.0298601)
Supplement: S1 Data — (ZIP) [file pone.0298601.s001.zip › Data/地级市Z_F工作报告.zip!/╜¡╬≈/╛░╡┬╒≥╩╨/└┤╫╘╣1⁄2╓┌║┼í╛┬φ┐╦╩2╛▌═°í┐_2006.docx]

各位代表：

　　现在，我代表市人民政府向大会作工作报告，请予审议。并请市政协委员和其他列席人员提出意见。

　　一、关于本届政府工作的回顾

　　本届政府自2001年选举产生五年多来，在省委、省政府和市委的正确领导下，全市广大干部群众深入贯彻党的十六大及十六届历次全会精神，以邓小平理论和“三个代表”重要思想为指导，树立和落实科学发展观，弘扬伟大的井冈山精神，围绕建设“经济重镇、旅游都市、特色瓷都”总体构想，积极探索景德镇由传统转向现代的发展新路，取得了改革开放和现代化建设的新成就，提前一年完成“十五”计划主要发展目标，全面超额完成“十五”时期各项任务，实施“十一五”规划开局良好。

　　全市生产总值由2000年的93.8亿元，增加到2005年的193.1亿元，按可比价计算，年均增长13.8%；财政总收入由2000年的8.24亿元增加到2005年的14.7亿元，年均增长12.3%；城镇固定资产投资总额由2000年的14.2亿元增加到2005年的88.4亿元，年均增长44.2%；社会消费品零售总额由2000年的28.1亿元增加到2005年的58.3亿元，年均增长15.7%；外贸出口总值由2000年的3642万美元增加到2005年的8781万美元，年均增长19.2%；城镇居民人均可支配收入由2000年的5096.6元增加到2005年的8779.4元，年均增长11.5%；农民人均纯收入由2000年的2379.4元增加到2005年的3589.4元，年均增长8.6%；人口自然增长率控制在7.94‰以内，比2000年下降1.9个千分点。

　　五年多来，政府工作取得的成绩主要体现在以下方面：

　　（一）开放型经济取得明显成效。按照对接长珠闽、融入全球化的思路，以实施大开放为主战略，以江浙沪闽粤、港澳台为招商重点，梯次承接产业转移，主攻工业项目、外资项目、重大项目，开放型经济成效明显。2001年至2005年，全市累计实际利用外资2. 11亿美元，外贸出口总值3.05亿美元，分别是“九五”时期的3.8倍、2.1倍；实际引进内资212亿元。其中，2003年实际利用外资1.03亿美元，比上年增长2.3倍，位居全省“四小市”总量第一、增幅第一、进度第一；2005年实际利用外资增幅和境外现汇率均居全省第一，外贸出口总值8781万美元，创历史最高水平。引进并实施了电化高科、海畅法蓝瓷、东璟陶瓷、阿古斯特直升机、立信药业等一批重大工业项目，国际500强企业——沃尔玛、国内500强企业——TCL集团相继落户我市。进一步加强了口岸建设，景德镇海关顺利开关。对外开放通道日渐改善，206国道洪源至杨家岭段建成通车，杭瑞高速景德镇段顺利通车，济广高速景鹰段建设迅速，南环高速公路建设加快实施，“十”字型高速公路主骨架初步形成。

　　（二）工业化进程快速推进。确立了“工业立市、工业兴市”的发展理念，提出工业为第一位发展战略的思路，先后出台了《关于进一步加快我市工业发展的若干意见》和《关于实施项目带动战略的意见》等一系列重要文件。大力推进工业化发展进程，设立了工业发展基金，实行了工业定期调度、定期交帐、重点项目领导挂点服务及重点新上工业项目检查督查等制度。工业投入快速增长，2001年至2005年，我市工业领域完成的固定资产投资额累计达89.73亿元，其中2005年当年完成投资28.95亿元，是2000年的4.7倍，预计2006年完成投资34亿元，比上年增长16%。通过实施一大批技改造项目，提高了企业技术创新水平，加快了企业信息化进程，促进了工业产业结构调整，增强了企业核心竞争力。工业总量不断提升，2005年，全市规模以上工业总产值完成159.2亿元，比2000年增长1.04倍，工业增加值完成49亿元，比2000年增长97.7%。预计2006年，规模以上工业增加值完成60亿元，比上年增长22%，其中地方工业实现高位增长，2005年完成工业增加值34.75亿元，比2000年增长2.62倍，占全市规模以上工业增加值的70.9%，比2000年提高35.4个百分点。工业企业利税较快增长，2005年，全市规模以上工业企业实现利税6.94亿元，上缴税收6.63亿元，分别比2000年增长24%和50.3%。至2006年底，工业主要经济指标基本实现超过全省平均水平的发展目标。

　　（三）陶瓷振兴取得重大进展。科技部批准实施部省共建景德镇国家陶瓷科技区域创新体系，高标准建设陶瓷产业、科研、人才、交流四大基地，为重振瓷都雄风建立了面向世界的国家级平台。景德镇陶瓷工业园区按照国家高新技术开发区的标准加快建设，13家高新技术陶瓷企业落户园区。高新区内的陶瓷园区1.5平方公里建成区初具规模，已有台资、美资及退城进园等40余家企业入园建厂。煤烧隧道窑拆除、燃气梭式窑整治工作全面推进，为陶瓷产业退城进园提供了有利条件。陶瓷产业结构不断调整优化，国有陶瓷企业改革稳步推进，民营陶瓷经济发展迅速。进一步整顿和规范了陶瓷展销活动，对国贸广场、广场南路、莲社南路、解放路等陶瓷市场进行了集中整治，加大了打击非法陶瓷展销力度，陶瓷销售市场秩序日益好转，取得了良好的经济和社会效益。陶瓷贸易交流成果显著，借部省之势，举全市之力，连续三年成功主办了主题突出、特色鲜明的景德镇国际陶瓷博览会，涵盖几十个国家和地区，呈现出起点高、规模大、人气旺、门类全、影响广、效益好等特点，在经贸洽谈、文化交流、技术合作等方面取得了丰硕成果，各项工作得到了部省领导的充分肯定。2005年，陶瓷工业总产值完成24.6亿元，比2000年增长90.7%；陶瓷出口创汇完成2184万美元，比2000年增长23.95%。预计2006年陶瓷工业总产值完成32亿元，比上年增长30%，出口创汇完成3500万美元，比上年增长60%，陶瓷产业在解困中发展，显现出新的发展生机和活力。

　　（四）农业经济稳步发展。按照统筹城乡发展的思路，坚持“少取”向“多予、放活”转变，取消了“三提五统”和除烟叶外的农业特产税，免征了农业税，实现了农民合同内“零”负担，促进了农业农村经济的发展，农业基础地位更加巩固。2005年实现农业总产值30.64亿元，按可比价计算，年均增长4.3%。农业基础设施建设进一步加强，完成了249座病险水库除险加固，增加旱涝保收农田4.5万亩，解决了9.38万人（头）的人（畜）饮水困难。农业综合生产能力明显提高，2005年粮食总产量达9.84亿斤，比2000年增长12.6%，年均增长2.4%。农业结构调整向深度和广度进军，农业经济实现了由以农为主向农业与林牧渔业、农产品加工业并驾齐驱的转变，全市逐步形成了“南菜北茶中间养”的生产格局，乐平、浮梁分别列为国家无公害蔬菜、茶叶示范基地县。农业产业化步伐加快，培育发展了1家国家级和10家省级产业化龙头企业，粮食、茶叶、蔬菜等主要产业初步走上了产业化发展道路，促进了现代农业的发展。农民收入实现了由务农收入为主向经营性、务工性收入为主的转变，从2004年起，连续三年实施“阳光工程”，培训农民1.86万人。全市外出务工人员占农村劳动力的45%。同时，以发展新产业、形成新机制、建设新村镇、树立新风尚、培育新农民、创建好班子为主要内容的社会主义新农村建设试点取得阶段性成果。农村公路建设成效显著，新建、改造农村公路1500公里，县乡通油（水泥）路达100%。农村生态建设、能源、村容村貌、人居环境等逐步改善，农村面貌发生了新变化。

　　（五）旅游产业日益壮大。围绕建设江南旅游都市，旅游产业实现跨越发展。全市旅游接待人次由2000年的122.01万人次发展到2005年的585.6万人次，年均增长36.9%。2006年预计接待675.9万人次，比上年增长15.4％。入境旅游人次由2000年的1.48万人次发展到2005年的4.81万人次，年均增长26.6%，预计2006年为5.92万人次，比上年增长23.2%。旅游总收入由2000年的4.53亿元发展到2005年的21.1亿元，年均增长36%。预计2006年为24.3亿元，比上年增长15.5%。旅游创汇由2000年的852万美元发展到2005年的1576万美元，年均增长13.1%，2006年预计1900万美元，比上年增长20.6%。旅游总收入占全市生产总值的比重由2000年的4.83%上升到2005年的10.9%。全市星级宾馆由2000年的4家发展到2005的24家；旅行社由2000年的3家发展到2005年的29家；旅游景区（点）由2000年的5个发展到2005年的12个；旅游车队从无到有发展到2005年的3家。具有国家导游员资格证的人员由11人发展到2005年的327人。创建了陶瓷历史文化博览区、高岭—瑶里风景区、浮梁古县衙景区3个国家AAAA级景区和我省唯一的全国工业旅游示范点——雕塑瓷厂。浮梁县获得中国优秀旅游名县称号，高岭—瑶里景区先后获得国家重点风景名胜区、国家自然与文化双遗产、中国历史文化名镇、国家森林公园、国家矿山公园和中国重点文物保护单位等称号。2002年，市政府从御窑原址迁出，使以御窑为中心的文物保护核心区得以完整保护。继去年成功创建中国优秀旅游城市之后，今年又被评为“中国最值得外国人去的50个地方之一”。全市旅游业带动直接就业人数7000余人，间接就业人数达3.5万余人，旅游业已逐步成为我市新的经济增长点和重要的支柱产业。

　　（六）城市面貌变化显著。 2001年以来，是城市建设投入最多、发展最快的时期，是城市面貌焕然一新的时期。五年来，全社会城市建设投资146亿元，是前50年投资总量的3.6倍；旧城改造拆迁面积215万平方米，是前50年拆迁总量的4倍；新建住宅面积260万平方米，是前20年建设总量的2.8倍；建设新区42平方公里，城市建成区扩大一倍。城市详规覆盖率由4%提高到88.2%，城市化率由35.1%提高到49.3%。城市框架整体拉开，高标准市区出入口大道已经建成，以昌南大道、景德大道、景东大道为轴线的西、南、东三块城市新区正在形成，西市区已初显山水生态文化特色。重点工程加快推进，城市功能设施日臻完善，开工建设了一江两岸及城市防洪工程、中国陶瓷博物馆、全国中小学陶艺培训中心、紫晶宾馆、西山湖宾馆、体育中心、财富大厦、白鹭大桥等36项重点工程，完成投资19.6亿元，其中17项工程竣工。旧城改造稳步推进，斗富弄和戴家弄街区改造等旧城改造工程进展顺利，人民公园改造成开放式公园，浮桥重建工程完工并投入使用，建成了人民广场等5个市民公园广场，建成了中国陶瓷城等5个专业市场。市政设施不断完善，5年来，城区主次干道全面建成改性沥青路面，城市道路总长由144公里提高到207公里，比2000年增长44%。自来水厂建成双回路高压线，日供水量由17万吨增长到30万吨。城市路灯数量3.3万盏，增长4.5倍，路灯亮化率达到98%。人均公共绿地面积由6.83平方米提高到9.5平方米，增长39%。绿化覆盖率、绿地率全部超过国家园林城市标准。新建李家220千伏等一批输变电工程，扩建了蛇垄220千伏等一批变电站。以弘扬陶瓷文化为主题的大型陶瓷装饰墙、文化墙、瓷灯柱和主题雕塑走上街头，提高了城市文化品位。人居环境明显改观，城市环境综合整治定量考核连续四年名列全省第一，被誉为江西最适宜人居的城市。城市创建成效显著，先后获得省卫生城市、省文明城市、省园林城市、中国优秀旅游城市、全国创建文明城市工作先进城市、国家园林城市等称号。景德镇告别了脏乱差的历史，市容市貌、基础设施、功能设施上了一个大台阶。

　　（七）各项改革深入推进。国有企业改革深入推进，全市209户市县属国有企业已完成改制108户，63户正在改制，改制面达82%。重点骨干企业改制工作取得突破性进展，黑猫炭黑股份公司成功上市，华意公司与TCL合作成功，战略重组取得较大进展。景德镇机场并入首都机场集团公司。建立健全国有资产监管机构，国有资产监督、管理、营运体系进一步完善。市城市信用社组建市商业银行工作进展顺利，农村信用合作社改革工作继续推进。交通投资体制改革迈出实质性步伐。农村税费改革取得突出成绩，各项配套改革稳步推进，农村综合改革开始启动，农村义务教育体制改革、水利管理体制工作改革和粮食流通体制改革顺利实施。林业产权制度改革扎实推进，林业资源得到较好的保护和培育。财政体制改革向前推进，健全了政府采购制，扩大了会计委派范围，开展了部门预算和国库集中支付试点工作。进一步理顺了财税收入关系，规范了财税征管秩序，建立健全了县乡财政发展激励机制。行政管理体制改革进一步深化，在巩固前两批行政审批事项清理成果的基础上，对行政许可项目、实施机关及收费项目进行了集中清理。

　　（八）社会事业全面进步。我省唯一一家国家级工程中心——国家日用及建筑陶瓷工程技术研究中心在我市正式组建运行，陶瓷研发创新能力大大增强。国家陶瓷质量监督检验中心被批准组建。国家知识产权局批准在我市设立的中国陶瓷知识产权信息中心正式建成并开通，国家知识产权试点城市各项工作进展顺利。江西省农产品生物保鲜工程技术研究中心组建并运行。新增国家级高新技术企业1家、省级高新技术企业7家，高新技术产业化综合指标连续四年名列全省第一，科技进步统计监测连续四年名列全省第二。教育事业蓬勃发展，义务教育率先在全省通过达标复查，高中阶段教育发展迅速，普通高考一、二本上线率、录取率、主体学科竞赛均居全省前列，职业教育、民办教育快速发展。老年教育富有成效，市老年大学被评为省老年大学示范校。景德镇陶瓷学院新校区首期工程基本建成，招生规模扩大，教学科研水平进一步提高。高校资源整合稳步推进，“景德镇学院”组建工作取得实质性进展。文化事业取得长足进步，大力实施文化精品工程，文艺创作繁荣发展，群众性文化活动蓬勃开展，对外文化交流进一步扩大。文物发掘保护开发取得新成绩，御窑遗址考古发掘被评为2003年度全国十大考古新发现。御窑遗址被国务院定为国家重点文物保护单位。陶瓷历史文化博览区被文化部命名为第二批国家文化产业示范基地、全国科普教育基地，景德镇手工制瓷工艺和传统瓷业营造技艺被列入《中国首批非物质文化遗产名录》。全面完成有线电视传输网改造，农村广播电视无线覆盖工程基本完成，自然村广播电视“村村通”工程顺利实施。2003年抗击非典取得重大胜利，初步建立并完善了公共卫生体系和疾病预防控制体系。认真做好乐平市和浮梁县新型农村合作医疗试点工作，全面完成农村卫生院上划县管工作。珠山区被命名为江西省社区卫生服务示范区。全民健身运动广泛开展，体育竞技水平在全省名列前茅，连续五届获省运会金牌总数前三名。关心青少年的健康成长，认真贯彻实施《未成年人保护法》，未成年人思想道德建设得到加强。社区建设逐步推进。普法依法治理工作成果显著，荣获2001年至2005年全国普法教育先进城市称号。国防后备力量建设和国防动员、双拥共建工作取得新进展，实现全国双拥模范城三连冠。新闻出版、计划生育、气象、水文、民族宗教、外事侨务、涉台事务、统计、档案、地方志、老龄、参事及妇女儿童等各项工作都取得了新成绩，全市人民的精神面貌发生可喜变化。

　　（九）人民生活水平大幅度提高。城乡居民收入增加，2005年，城镇居民人均可支配收入和农民人均纯收入分别比2000年增长72.2%、50.9%。城乡居民居住条件明显改善，城镇居民人均房屋使用面积达20.37平方米，农村居民人均住房面积达37.75平方米。向符合条件的困难家庭提供了一批廉租住房，向低保特困人员发放廉租补贴32万元，新开工建设100套廉租住房。就业和再就业工作稳步推进，五年来全市新增城镇就业人员5.81万人；至2005年底，全市跨地区劳务输出17.43万人次。社会保障制度进一步完善，企业离退休人员基本养老金按时足额发放率和社会化发放率100 %，城镇职工基本养老保险、基本医疗保险、失业保险、工伤保险等覆盖面进一步扩大，社会保障水平不断提高。城镇居民最低生活保障标准数次提高，已达到每月170元，其中“三无”人员 190元，位居全省第二。农村居民最低生活保障制度全面启动。对城乡困难群众的社会救助体系进一步健全，救助对象扩大，救助标准提高。安全生产工作有效加强，重大安生生产事故大幅下降。高度重视信访工作，进一步畅通信访渠道，积极处理群众来信来访，着力处理并解决了一批信访突出问题。全面推进社会治安综合治理，努力构建和谐平安瓷都，社会政治局面持续稳定，荣获2001年至2004年全国社会治安综合治理先进城市称号。

　　（十）政务环境进一步优化。依法行政水平进一步提高，认真执行市人大及其常委会的决议、决定，自觉接受人大及其常委会的法律监督和工作监督，自觉接受政协和各民主党派、工商联的民主监督，自觉接受人民群众的社会监督和新闻媒体的舆论监督。五年多来，共办理人大代表意见、建议349件，政协提案1330件。行政行为逐步规范，深入实施《行政许可法》和《全面推进依法行政实施纲要》，制定重大政策坚持征求意见、法制论证和集体决策制度，行政决策进一步科学化、制度化、民主化、法制化。相继出台了《景德镇市重点工程建设项目管理办法》等一系列规范性文件，对重点工程建设、土地、房地产、税费减免等工作实行规范化管理，做到了有规可循、有章可依。行政服务措施进一步完善，清理、压缩行政审批事项549项，对保留的行政许可项目面向社会公开。加强行政服务中心建设，行政许可项目联合集结审批，推行“一门受理、抄告相关、并联审批、限时办结”运行方式。不断优化发展环境，成立了行政投诉中心，全面开展政务环境评议评价活动和投资环境集中整治活动，组织部分市人大代表、政协委员及人民监察员、特邀监察员对市直部门和单位优化投资环境情况进行测评，努力营造“亲商、富商、安商”氛围。

　　2006年是“十一五”规划的开局之年，在全面超额完成“十五”计划确定的各项目标任务的基础上，各项工作加快推进，经济继续保持良好发展态势，各项社会事业取得新的长足进步。2006年，预计全市实现生产总值224亿元，比上年增长14.6%；财政总收入16亿元，增长9.3%，其中一般预算收入9.37亿元，增长11.1%；城镇固定资产投资89亿元，增长0.6%；社会消费品零售总额68.1亿元，增长16.8%；外贸出口总值1亿美元，增长22.7%；实际利用外资5600万美元，增长10%；实际利用内资48.8亿元，增长15%；城镇新增就业人员2.1万人，城镇登记失业率3.4%；城镇居民人均可支配收入10120元，增长15.3%；农民人均纯收入3960元，增长10%；人口自然增长率控制在7.76‰以内。与此同时，各项社会事业取得了新的成绩。

　　各位代表，回顾五年多来的发展历程，我们克服重重困难，成绩来之不易，这是省委、省政府和市委正确领导，市人大依法监督和市政协参政议政，全市人民辛勤劳动的结果。在此，我代表市人民政府，向全市广大工人、农民、知识分子、干部和离退休老同志，向各民主党派、无党派、工商联、人民团体和社会各界人士，向驻市解放军、武警官兵和公安干警，向来景的投资者和外来劳动者，向所有参与、关心和支持瓷都发展的海内外友好人士表示衷心的感谢，并致以崇高的敬意！

　　过去五年多来，虽然我们取得了很大的成绩，但发展中也存在一些困难和问题，主要是：经济总量仍然偏小，开放程度还不高，部分经济指标在全省排位靠后，就业和社会保障水平低，部分群众生活较困难，影响稳定的因素仍然存在，政府自身建设有待于进一步加强等。对于这些困难和问题，我们将高度重视，并在今后的工作中努力克服和解决。

　　二、关于《景德镇市国民经济和社会发展第十一个五年规划纲要（草案）》的说明

　　2006年，我们已经进入了第十一个五年规划时期。站在新的起点上谋划今后五年的发展，我们对未来更加充满信心。根据《中共景德镇市委关于制定全市国民经济和社会发展第十一个五年规划的建议》，市政府制定了《景德镇市国民经济和社会发展第十一个五年规划纲要（草案）》（以下简称《纲要（草案）》），提交本次大会审议。

　　《纲要（草案）》坚持以科学发展观统领经济社会发展全局，着眼加快崛起和富民兴市，总结过去，展望未来，在全面分析今后发展面临的国内外环境的基础上，提出了今后五年全市经济社会发展的总体要求、奋斗目标和主要任务，描绘了全市发展的宏伟蓝图和美好前景。《纲要（草案）》提出，“十一五”时期是我市经济社会发展的重要战略机遇期，也是全面建设小康社会的关键时期。要实现建设“经济重镇、旅游都市、特色瓷都”的战略构想，打造富裕、民主、文明、和谐、美丽的新瓷都，就必须从我市经济欠发达、经济规模小的实际出发，切实增加投入，加速做大总量，夯实经济基础，全面提升综合实力，在新的发展起点上开创我市社会主义经济建设、政治建设、文化建设和社会建设新局面。

　　——经济实力显著提高。到2010年，全市生产总值达到390亿元，年均增长13%，比2005年翻一番；财政总收入达到30亿元，年均增长15.3%，比2005年翻一番；规模以上工业增加值达到122亿元，年均增长20%；全社会固定资产投资达到220亿元，年均增长20%。

　　——经济结构继续优化。到2010年，三次产业比例将由2005年的10.01：52.65：37.34调整到6∶55∶39，农业现代化水平进一步提高，非农就业比重达到55%左右，工业增加值占生产总值的比重达到53%，服务业水平大幅提升，所有制结构、产业结构、企业组织结构、技术结构和产品结构进一步改善。

　　——经济增长质量明显提高。到2010年，资源利用效率显著提高，单位生产总值能耗比“十五”期末降低20%，单位工业增加值用水量降低30%，主要污染物排放总量减少10%，全社会研究与开发经费占全市生产总值的比重达到1.2%，科研成果向现实生产力转化加快，科技进步对经济增长的贡献率达到50%以上，财政总收入占生产总值的比重稳步提高。

　　——统筹城乡发展取得重大进展。到2010年，景德镇市城区人口达到60万人，城镇化率达到56%以上，城镇布局更加合理，城镇功能全面提升，中心城市综合竞争力和辐射带动能力增强，建设社会主义新农村取得明显成效。

　　——基础设施继续完善。建设快速便捷的综合交通运输体系、安全可靠的能源保障体系、防洪减灾和开发利用并重的水利保障体系、优质安全的信息化服务体系，以满足经济社会发展及人民生活需要。

　　——公共服务体系更加健全。确立“教育强市”发展目标，到2010年，全面实现义务教育普及提高目标，高中阶段毛入学率达到75%，力争80%，普高、职高招生比例相当，城镇居民平均受教育年限达到8.5年，食品安全监测抽检合格率达到95%，每千人口医院和卫生院床位数达到2.5张，社会保障体系比较健全，城镇职工参加基本养老保险、基本医疗保险和失业保险覆盖率分别达到91%、75%、90%。

　　——可持续发展能力增强。到2010年，森林覆盖率达到62%，景德镇城市建成区绿化覆盖率达到45%以上，城市空气质量稳定在国家Ⅱ级标准，市县集中式饮用水源地水质达标率达到100%，重点工业污染源排放达标率达到100%，城市污水集中处理率达到80%，城市垃圾无害化处理率达到90%，工业固体废物处置利用率达到98%以上，人口自然增长率控制在8‰以内。

　　——人民群众生活进一步改善。到2010年，城镇居民人均可支配收入达到14138元，农民人均纯收入达到5081元，城镇居民人均住宅建筑面积达到30平方米，城市居民人均绿地面积不少于9平方米，城镇登记失业率控制在4.5%以内，五年内新增城镇就业人员10万人。

　　——市场经济体制比较完备。行政管理、国有企业、财税、金融、科技、教育、文化等领域的改革和制度建设取得突破。开放型经济达到新水平，实际利用外资年均增长10%，外贸出口总值年均增长20%，到2010年，分别达到8200万美元和2.1亿美元。

　　实现上述目标，必须以邓小平理论和“三个代表”重要思想为指导，全面贯彻落实科学发展观，紧紧抓住重要战略机遇期，着眼建设经济重镇、旅游都市、特色瓷都，增加投入，做大总量，夯实基础，立足科学发展，推进自主创新，完善体制机制，促进社会和谐，大力推进农业农村现代化、新型工业化、新型城镇化、经济国际化和市场化，建设创新创业瓷都、绿色生态瓷都、和谐平安瓷都，建设独具山水生态文化特色的最适宜人居的城市，进一步提升发展质量和层次，增强全市经济社会综合实力，为景德镇重振雄风再创辉煌、全面建设小康社会打下坚实的基础。

　　第一，加快社会主义新农村建设，扎实推进农业农村现代化。建设社会主义新农村，是推进农业和农村现代化的必由之路，对于站在新的历史起点上实现全市经济社会又快又好发展，具有十分重要的意义。《纲要（草案）》提出，“十一五”期间要以促进农业发展、农民增收、农村和谐为重点，以农村经济社会全面发展为目标，扎实推进社会主义新农村建设。要以发展农村生产力为中心，贯彻“希望在山、潜力在水、重点在田、后劲在畜、出路在工”的战略决策和“扬优成势重特色、开放发展富农民”的发展思路，全面提高农业发展水平。加快农业科技示范园区建设步伐，把其真正打造成为我市农业对外开放的主战场，成为农业科技创新的“孵化器”。到2010年，全市农业科技贡献率达到45%。进一步巩固和提高粮食生产能力，粮食综合生产能力稳定在10亿斤以上。加强市级储备粮制度建设，确保粮食安全。不断调整优化农业结构，重点发展壮大蔬菜、茶叶、养殖、绿色森林食品等特色产业，积极推进“一村一品、一县一业”。加快农业产业化步伐，把农业、工业、三产建成复合型高效产业，力争五年内年销售收入5亿元以上的龙头企业达到4家，1亿元以上的达到7家，5000万元以上的达到20家。以促进农民增收、财政增长、壮大经济实力为主要任务，充分发挥各县（市、区）经济、生态、区位等比较优势，打造各具特色的经济板块。加强农村基础设施建设，贯彻工业反哺农业、城市支持农村的方针，加快基础设施建设向农村延伸步伐，五年内新建和改造农村公路1588公里，实现县至乡镇通三级以上公路，乡至行政村通沥青（水泥）路率达到100%，解决农村12万人口的饮水安全问题，使农村自来水普及率达到55%。加强农村公共服务，巩固农村九年制义务教育，建立农村义务教育投入保障机制，建立基本设施齐全的农村卫生、计生服务网络，继续抓好农民培训工作，培养有文化、讲道德、守法纪、懂技术、会经营的新型农民。加快推进农村综合改革，继续深化农村土地承包制、农村金融体制、粮食流通体制、水利管理体制、林业产权体制等改革。加强农村精神文明建设，推进农村民主化管理，大力倡导文明新风。

　　第二，转变经济增长方式，走新型工业化道路。“十一五”时期，是我市加速工业化进程的关键时期，也是经济结构加速转型的重要时期，我市工业既要突出解决发展规模问题，也要解决增长质量问题。《纲要（草案）》提出，要深入实施以新型工业化为核心的发展战略，进一步发挥工业化在统筹城乡发展、解决群众就业、建设经济重镇中的主导性作用。把工业化与信息化、生态化结合起来，大力推动区域科技创新，改造提升传统产业，推进产业技术升级。以市场化手段积极推进优势资源的整合，同时，重点实施YK5新车、民用直升机和干熄焦生产线等一批重大工业项目，培育一批主业突出、核心竞争力强、带动作用大的大企业、大集团，不断发展壮大机械家电、航空汽车、化工医药、电子信息、电力能源、新型陶瓷和特色食品等七大支柱产业。争取到“十一五”期末，机械家电产业总产值达到60亿元；航空汽车产业总产值达到110亿元；化工医药产业总产值达到80亿元；电子信息产业总产值达到25亿元；电力能源产业总产值达到25亿元；新型陶瓷产业总产值达到30亿元；特色食品产业总产值达到10亿元。加快工业园区建设，围绕基础设施、招商引资、产业聚集、高效服务四个重点，着力提升园区集约化发展水平、产业带动能力、就业吸纳能力和对当地经济发展的贡献率，重点打造景德镇高新技术产业园区、景德镇陶瓷工业园区、乐平工业园区三大园区，力争到2010年末，三个园区销售收入均突破100亿元。坚持走资源节约型、环境友好型的路子发展工业，加强资源节约和综合利用工作，努力做到工业资源能源消耗水平与可持续发展相适应，工业大发展与环境承载能力相适应，工业增长方式与广大群众对环境的期望相适应。

　　第三，把握历史机遇，全力推动陶瓷产业振兴。陶瓷是立市之本，称都之源。始终把陶瓷放在特殊重要的地位，打响瓷都品牌，重振瓷都雄风，是我们义不容辞的历史责任。因此《纲要（草案）》着重提出，要以实现陶瓷产业基本振兴为目标，努力打造产学研一体化、产供销市场化国际化的大陶瓷产业新格局，建设名副其实的“中国的瓷都、世界的景德镇”。要多方并举，加快培育发展一批现代陶瓷企业，实现产业集成，做大总量。依托国家陶瓷科技城四个基地和国际瓷博会等国家级平台，突出抓好陶瓷招商引企，承接国际陶瓷优强企业产业转移，引进国际知名企业；加快国有、集体陶瓷企业的改制重组，重点支持一批有后劲、有潜力的民营企业成为上规模、上档次、有竞争力的龙头企业，引导已经完成原始积累的小型作坊或划小承包的企业实施管理重组，发展新型企业。要依靠科技，全面提升陶瓷产业层次。以提高陶瓷自主创新能力为中心环节，整合陶瓷教育科研资源，加强陶瓷创新体系建设，实施陶瓷创新工程。充分发挥国家日用及建筑陶瓷工程技术研究中心等科研机构作用，加速陶瓷科研成果向现实生产力转化，改造传统陶瓷生产，大力发展高档日用瓷、建筑卫生陶瓷、高压电瓷电器等高新技术陶瓷产业，构筑后发优势，为陶瓷产业振兴提供有力的支撑。要大力实施名牌战略，整顿和规范陶瓷市场。加强“景德镇”证明商标的管理和保护，严厉打击假冒伪劣等侵权行为。进一步整顿和规范陶瓷出国展销等市场秩序，培育一个集天下名瓷之大成的陶瓷市场，并连接国内外主要城市，打造国际化、市场化的陶瓷集散地和物流中心，加快推进陶瓷贸易国际化进程，以流通拉动生产，到2010年，培植一批年外贸出口总值超千万美元的陶瓷企业。加强陶瓷产业基础建设，强化产业振兴保障。实施陶瓷原料标准化建设工程，加紧陶瓷原料勘探和基地建设，打造资源保障体系。实施燃气基地建设工程，引进高效天燃气，降低生产成本。实施陶瓷窑炉改造工程，提升模具、窑炉、耐火材料水平。更新企业机械装备，提高花纸金水供应水平和陶瓷包装生产质量。到2010年，实现高新技术陶瓷销售收入达到25亿元，日用陶瓷销售收入达到30亿元，艺术瓷销售收入达到25亿元，建筑卫生陶瓷销售收入达到5亿元。

　　第四，弘扬陶瓷文化，发展壮大旅游产业。发展旅游产业，瓷都具有得天独厚的人文优势，目前旅游产业已逐步成为我市新的经济增长点和重要的支柱产业。为此《纲要（草案）》强调，旅游经济是我市开放型经济的重要组成部分，必须把旅游作为富民兴市的重点产业，坚持以大开放的理念、大开放的战略、大开放的手段来促进新一轮的旅游大发展，实现发展水平的全面跨越。实施旅游十大工程，构筑以陶瓷历史文化为主线的大旅游格局，实现旅游接待人次、旅游收入翻番目标，使景德镇真正成为历史文化与现代文明融为一体的江南旅游都市。坚持保护为先、合理利用的原则，保护好历史文物，打造城区里弄景点，将文物资源开发转化为旅游资源和经济增长点，展示历史文化名城的古韵风貌。以陶瓷旅游为主线，大力发展文化旅游、生态旅游、工业旅游。统筹规划景区景点建设，整合与发掘旅游资源，打造出规模性、震撼性、特色性、唯一性的瓷都旅游品牌，提升瓷都特色旅游形象。在已有3个国家AAAA级景区基础上再创建御窑遗址、洪岩仙境等3-5个国家AAAA级景区和1个国家AAAAA级景区。实施旅游管理体制改革，创新旅游营销机制，创新发展机制。通过完善交通、住宿、娱乐、购物、餐饮和游客集散等服务设施，提升旅游配套服务功能。加强旅游市场横向联合，充分利用“十”字型高速公路主骨架初步形成的机遇，加快融入长三角、珠三角、京津唐及长江沿线旅游网络，构建“六山两湖”之间的精品旅游线路，积极开拓东南亚、日韩等国际旅游市场。

　　第五，统筹城乡发展，推进新型城镇化。《纲要（草案）》强调，我市将按照城市向西扩展，工业向南聚集的规划，以中外闻名的瓷都为特色，面向世界、开放创新，建设山水、生态、文化特色城市，把景德镇打造成为赣浙皖三省交界区域经济实力强、城市功能优、人居环境好的中心城市。要按照社会管理、经济管理、规划管理、公共建设管理和生态环境管理五大原则，积极实施城市管理，加大城市建设力度。积极打造“一城三区九大功能组团”的城市发展格局，着力将包括生态路在内的昌南组团、洪源组团和罗家组团建设成集金融、居住、休闲娱乐为一体的生态新区。加快城市基础设施、功能设施建设，在保护历史文物、保持历史风貌的基础上，使城市更加人性化、便捷化。加快壮大物流业、商贸业、房地产业，尤其要进一步完善经济适用房、廉租房制度，有计划地新增经济适用房，让中低收入家庭买得起基本住房。持续深入地开展创建工作，争创国家生态园林城市、国家卫生城市、国家环保模范城市、全国文明城市、中国最佳优秀旅游城市。同时，《纲要（草案）》强调，要按照产业集聚、功能完善、节约土地、集约发展、合理布局、各具特色的原则，统筹城乡空间布局，加快形成合理的城市等级规模结构，积极稳妥地推进城镇化进程。按照我市区域地理环境和产业分布的特点，完成市域城镇体系规划，形成规模适度、布局合理、各具特色、整体协调的城镇体系。以县域工业为核心，突出打造特色经济，使县域经济总量、产业结构、城乡居民收入、财政收入、农村劳动力转移和城镇建设提高到新的水平。

　　第六，加大改革攻坚力度，加快经济市场化。目前，改革正处于攻坚阶段，要继续解放思想，勇于探索，大胆突破，敢于攻坚，统筹推进各项改革，提高经济市场化水平。《纲要（草案）》提出以转变政府职能和深化国有企业、财税、投融资等改革为重点，加快完善社会主义市场经济体制，力争在一些重要领域和关键环节取得突破。要更加注重改革的总体指导和统筹协调，更加注重把行之有效的改革措施科学化、规范化、制度化和法制化。进一步深化行政管理体制改革，推进政府管理创新，转变政府职能，提高政府管理和服务水平，特别是要把工作重点转到营造良好发展环境、提供优质高效服务、维护社会公平正义上来，建设勤政、务实、廉洁、高效的政府。进一步健全国有资产监督管理体制和国有资本有进有退、合理流动的机制，特别是注重整合盘活国有资产，加速国有资产从竞争性领域退出。加快推进国有企业改革，规范改制行为，防止国有资产流失，并着力化解改制矛盾。加大股份制改革力度，培育一批有特色、有潜力的上市企业后备队伍，争取到2010年新上市企业3家，改组或新建股份有限公司5家。鼓励各类所有制经济特别是非公有制经济的发展，进一步放手发展民营经济，放宽市场准入，为各类投资主体创造公平、公开、公正的法制环境、政策环境和市场环境。完善土地、技术和资本市场，建立城乡统一的劳动力市场，发展中介服务组织，健全市场监管机制。积极推进财税各项改革，坚持依法治税，建立健全公共财政体系，加强财政管理，提高理财水平，增强政府调控能力。深化投融资体制改革，推进投资主体和产权多元化，特别是要坚持有所为有所不为，合理界定政府投资范围，更大程度地发挥市场对资源配置的基础性作用。多方引进不同所有制金融机构，促进地方中小金融企业发展，扩大金融市场规模，完善金融服务体系，进一步加大金融资金对地方经济的投入。

　　第七，提高对外开放水平，加快推进经济国际化。推进经济国际化，既是进一步拓展发展空间的需要，也是“十一五”期间经济社会发展的强大动力。要坚持“引进来”和“走出去”相结合，利用好国际国内两个市场、两种资源，充分发挥欠发达地区的后发优势，开拓我市经济的发展空间。《纲要（草案）》强调，要坚持对外开放与对内开放并举，以“对接长珠闽，融入全球化”为导向，全面提升开放型经济发展水平。充分发挥我市的区位优势、资源优势和后发优势，加强对接与协作，积极争取国家和省重点项目的支持，争取引进国有大型企业，更多地吸纳沿海地区的产业转移。积极开展与国际友好城市的合作与交流，拓宽合作领域。拓展基础设施、公共服务设施、社会事业招商，着力引进大型龙头项目。提高利用外资水平，实现项目高位嫁接，优化招商引资结构，创新招商方式方法，积极吸引国外、境外、省外、市外的资本、技术及其他各类要素进入我市。鼓励引导现有客商增资扩股，提高已落户企业对我市经济增长的贡献率。实施积极的对外贸易鼓励政策，进一步完善外贸促进服务体系，做大对外贸易规模，优化出口产品结构，转变对外贸易增长方式，到2010年，力争年出口创汇500万美元以上的企业达到12家，其中年出口创汇1000万美元以上的达到4家。创新服务机制，打造服务经济社会发展、提供高效政务服务的平台，提高办事质量和效率，营造良好的投资环境。加强口岸建设，推进铁路、公路、水路联运，增强铁路、公路、水路口岸作业区功能，开辟航空口岸通道。

　　第八，坚持以人为本，着力建设“创新创业瓷都、绿色生态瓷都、和谐平安瓷都”。《纲要（草案）》提出，“十一五”期间，要在科技重点领域、科技发展水平、科技成果转化方面取得突破，增强自主创新能力，不断完善区域创新体系，确立企业在技术创新和科技投入中的主体地位，使科技进步综合水平达到全省前列，力争到2010年高新技术产业增加值占工业增加值比重达35%，科技进步对经济增长贡献率达50%以上。推进创新创业瓷都建设。坚持教育优先发展战略，深化教育管理体制改革，强化政府对义务教育的保障责任，全面加强素质教育，提高教育教学质量，促进基础教育、职业教育、高等教育、民办教育、老年教育等各级各类教育协调发展，构筑终身教育体系。牢固树立人才资源是第一资源的观念，大力加强人才队伍和人才市场建设，营造良好的人才环境，不断完善用人机制，扩大人才队伍总量，优化人才队伍结构。进一步营造创业文化，培育创业主体，拓宽创业领域，优化创业环境，大力推动全民创业。

　　瓷都良好的生态环境，是我们的宝贵财富。《纲要（草案）》强调，推行有利于资源节约和环境保护的生产模式、消费模式和城乡建设模式，积极发展循环经济，建设资源节约型、环境友好型的绿色生态瓷都。按照保护、恢复、建设、治理相结合的原则，从事后治理向事前保护转变，从人工建设为主向自然恢复为主转变，进一步改善生态环境。坚持预防为主，防治结合，切实保护好绿水青山。推行清洁生产、清洁建设、清洁消费，进一步改善环境质量。按照“减量化、再利用、资源化”的原则，探索发展循环经济的有效模式，实现由资源—产品—废弃物的单向线性过程，向资源—产品—废弃物—再生资源的反馈式循环过程转变。

　　在实施“十一五”规划的过程中，要按照以人为本的要求，更加注重社会发展，更加注重社会公平，突出解决好人民群众最关心、最直接、最现实的切身利益问题。进一步加强社会治安防控体系建设，全面落实社会治安综合治理各项措施，切实推进和谐平安瓷都建设。加强国防后备力量建设和国防动员工作，进一步提升双拥共建水平。为此，《纲要（草案）》从完善社会保障体系、扩大就业、发展文化事业、提高人民群众健康水平、加强安全生产、维护社会稳定、加强社会主义精神文明、民主政治建设和国防建设等方面作出了全面部署，为顺利实施“十一五”规划提供可靠保证。

　　第九，加强基础设施建设，为加快经济发展提供有力支撑。基础设施是经济社会发展的重要物质基础和平台，是经济社会各个环节正常运转和协调发展的先决条件。《纲要（草案）》指出要坚持统筹规划、合理布局、量力而行、适度超前的原则，进一步加大投资力度，推进交通、能源、水利、信息基础设施建设，使基础设施保障能力与经济发展水平相适应。按照客运快速化、货运物流化的要求，优化交通结构，加强各种运输方式相互衔接和协调发展，发挥组合效率和整体优势，基本形成较为完善的交通运输体系，全面提升综合交通能力。完成景鹰高速、南环高速公路建设项目，争取九景衢铁路立项开工。坚持节能优先、煤为基础、多点发展、合理布局、保障安全的方针，加强以电力为中心的能源建设，积极开发利用新能源和可再生能源，优化能源生产和消费结构，促进能源可持续发展和有效利用，构筑稳定的能源供应体系。新建涌山、南山220千伏以及马鞍山、湘湖110千伏等输变电工程，继续完善城市中低压供电网络。坚持突出重点，完善防洪减灾体系和水资源开发利用体系，保障人民群众生命财产安全，满足生产生活需要。加快信息技术在经济社会各领域的普及应用，提高经济社会运行质量和效率。实行重点突破、以点带面、全面推进，加速政务信息化、企业信息化、公共服务信息化。

　　各位代表，新的历史征程呼唤着我们，新的宏伟目标激励着我们，让我们紧密团结在以胡锦涛同志为总书记的党中央周围，高举邓小平理论和“三个代表”重要思想伟大旗帜，全面贯彻落实科学发展观，在省委、省政府和市委的正确领导下，认真贯彻落实市第九次党代会精神，以更加饱满的精神状态、更加务实的工作作风，继往开来，与时俱进，团结拼搏，扎实工作，为创造富裕、民主、文明、和谐、美丽的新瓷都，实现建设“经济重镇、旅游都市、特色瓷都”的新跨越作出更大贡献！
